# Vitamin D Receptor Gene and Aggrecan Gene Polymorphisms and the Risk of Intervertebral Disc Degeneration — A Meta-Analysis

**DOI:** 10.1371/journal.pone.0050243

**Published:** 2012-11-28

**Authors:** Ge Xu, Qiang Mei, Daijun Zhou, Jinlin Wu, Luo Han

**Affiliations:** 1 Department of OrthoPedics, Southwest Hospital, Third Military Medical University, Chongqing, China; 2 4th team of Cadet Brigade, Third Military Medical University, Chongqing, China; 3 Department of Endocrinology and Metabolism, The Second Affiliated Hospital of Chongqing Medical University, Chongqing, China; 4 Department of High Altitude Diseases, College of High Altitude Military Medicine, Third Military Medical University, Chongqing, China; National Central University, Taiwan

## Abstract

**Background:**

A series of studies have been conducted to evaluate the associations between vitamin D receptor (*VDR*) and aggrecan variable numbers of tandem repeat (*VNTR*) polymorphisms and the risk of intervertebral disc degeneration (IDD), but produced conflicting results.

**Objective:**

we performed a meta-analysis to address a more accurate estimation of the associations between the above gene polymorphisms and the risk of IDD.

**Methods:**

A comprehensive literature search was conducted to identify all the relevant studies. The fixed or random effect model was selected based on the heterogeneity test among studies evaluated using the *I* ^2^. Publication bias was estimated using Begg's funnel plots and Egger's regression test.

**Results:**

A total of 9, 5, 3, and 7 studies were finally included in the analyses for the associations between the *VDR TaqI* (rs731236), *FokI* (rs2228570), *ApaI* (rs7975232), or aggrecan *VNTR* polymorphisms and the risk of IDD, respectively. The combined results showed that none of the *VDR* (*TaqI, FokI, ApaI*) polymorphisms were significantly associated with the risk of IDD. In contrast, the alleles with shorter *VNTR* length was found to significantly increase the risk of IDD (≦25 *vs.* >25: OR = 1.850, 95%CI 1.477–2.318; ≦23 *vs.* >23: OR = 1.955, 95%CI 1.41–2.703). Subgroup analysis confirmed the above results. After excluding studies deviated from Hardy-Weinberg equilibrium (HWE) in controls, no other studies were found to significantly influence the pooled effects in each genetic model. No potential publication bias was detected.

**Conclusion:**

This meta-analysis suggested that the alleles with shorter *VNTR* length significantly increased the risk of IDD, while the *VDR* (*TaqI, FokI, ApaI*) gene polymorphisms were not significantly associated with the risk of IDD. Since potential confounders could not be ruled out completely, further studies are needed to confirm these results.

## Introduction

Intervertebral disc degeneration (IDD) is a major pathological process implicated in low back pain, and is a prerequisite to disk herniation [1]. IDD has been attributed to the accumulation of environmental factors, primarily mechanical insults and injuries, imposed on the “normal” aging changes [2]. However, epidemiological studies on families and twins have suggested that inheritance may be the major determinant of IDD [3]–[5]. So far, several gene polymorphisms have been demonstrated to be associated with the risks of IDD [6].

Vitamin D receptor (*VDR*) gene is the first reported gene potentially associated with IDD risks [7]. *VDR* gene is located on human chromosome 12 (12q12–q14), with a length of 100 kb, and has more than 100 restriction endonuclease cutting site polymorphisms [8]. *VDR* is a member of the steroid superfamily of nuclear receptor, which plays a key role in the regulation of the transcriptional activity of vitamin D metabolite, 1α, 25-dihydroxyvitamin D3 [9]. *VDR* gene polymorphisms are thought to contribute to a variety of disorders including osteoporosis, osteoarthritis, tumor, and cardiovascular diseases [6]. In the past decades, there has been increasing interest in the study of the association between *VDR* gene polymorphisms and the risk of IDD. These studies have mostly focused on a few selected variants, including the *TaqI* (rs731236), *FokI* (rs2228570), and *ApaI* (rs7975232) restriction sites. However, the results have been inconsistent. Some studies suggested that *VDR TaqI* gene polymorphism was associated with increased risk of IDD [10]–[12], while others showed no association [12], [13], and even associated with reduced risk of IDD [14].

Aggrecan is a large aggregating proteoglycan which is a functionally important component of intervertebral disc and articular cartilage. Humans are known to uniquely exhibit variable numbers of tandem repeat (*VNTR*) polymorphism within the aggrecan CS1 domain [15]. The association between aggrecan *VNTR* polymorphism and the risk of IDD has been investigated in several recent studies. Kawaguchi *et al.* firstly reported that subjects with shorter *VNTP* length of the aggrecan had a risk of having multilevel IDD [16], which was supported by the studies by Solovieva [17], Cong [18], and Mashayekhi [19]. However, the studies by Roughley [20] and Noponen-Hietala [21], showed no such association.

As mentioned above, the associations between *VDR* and aggrecan polymorphisms and the risks of IDD have been investigated in a series of studies, but obtained conflicting results. Race, age, occupation, etc may have introduced variability into the test of genetic susceptibility to disease in the different studies. Thus, we performed a meta-analysis from all eligible studies, in order to provide more accurate estimate of the association of the above gene polymorphisms and the risk of IDD.

## Materials and Methods

### Literature and Search Strategy

A computerized literature search was conducted for the relevant available studies published in English or Chinese from 5 databases including PubMed, ISI Web of Science, China National Knowledge Infrastructure (CNKI), Database of Chinese Scientific and Technical Periodicals (VIP), and China Biology Medical literature database (CBM). The search strategy to identify all possible studies involved use of combinations of the following key words: (“vitamin D receptor” or “VDR” or “aggrecan”) and “polymorphism” and “disc degeneration”. The reference lists of review articles, clinical trials, and meta-analyses were also hand-searched for the collection of other relevant studies. If more than one article were published using the same case series, only the study with largest sample size was selected. The literature search was updated on May 1, 2012.

### Inclusion Criteria

The studies included must meet the following criteria: (1) evaluating the associations between *VDR* (*TaqI, FokI, or ApaI*) polymorphisms or aggrecan *VNTR* polymorphism and the risk of IDD; (2) case-control or cohort design; (3) providing sufficient data for calculation of odds ratio (OR) with the corresponding 95% confidence interval (95%CI). When genotype frequencies and OR with 95%CI were all not available, authors were contacted to request the relevant information. All identified studies were carefully reviewed independently by two investigators to determine whether an individual study was eligible for inclusion in this meta-analysis.

### Data Extraction

Data were extracted independently by two investigators who reached a consensus on all of the items. The following information was extracted from each study: (1) name of the first author; (2) year of publication; (3) country of origin; (4) ethnicity of the study population; (5) source of control subjects; (6) numbers of cases and controls; (7) gender and age of enrolled subjects; and (8) numbers of genotypes in cases and controls.

### Statistical Analysis

We use χ2 analysis with exact probability to test departure from Hardy-Weinberg equilibrium (HWE) for the genotype distribution. The associations of four gene polymorphisms with IDD were estimated by calculating pooled ORs and 95%CI. The significance of the pooled effect size was determined by *Z* test. Heterogeneity among studies was assessed using Q test as well as the *I^2^* statistic, which was documented for the percentage of the observed between-study variability due to heterogeneity rather than chance [22]. The DerSimonian and Laird random effect model (REM) was used as the pooling method when *I^2^*>50%, otherwise, the Mantel-Haenszel fixed effect model (FEM) was considered to be the appropriate choice [22]. Subgroup analyses were stratified by ethnicity, gender and age. Cumulative meta-analysis was performed to assess whether the combined estimate changed in the same direction over time [23]. Influential analysis was undertaken by removing an individual study each time to check whether any of single study could bias the overall estimate [24]. An individual study was suspected of excessive influence, if the point estimate of its omitted analysis lies outside of the 95%CI of the combined analysis. Begg's funnel plots and Egger's regression test were undertaken to assess the potential publication bias [25]. Probability less than 0.05 was judged significant except for the *I* ^2^ statistic. Data analysis was performed using STATA version 11 (StataCorp LP, College Station, Texas, USA).

## Results

### Characteristics of Studies

**Figure 1:**
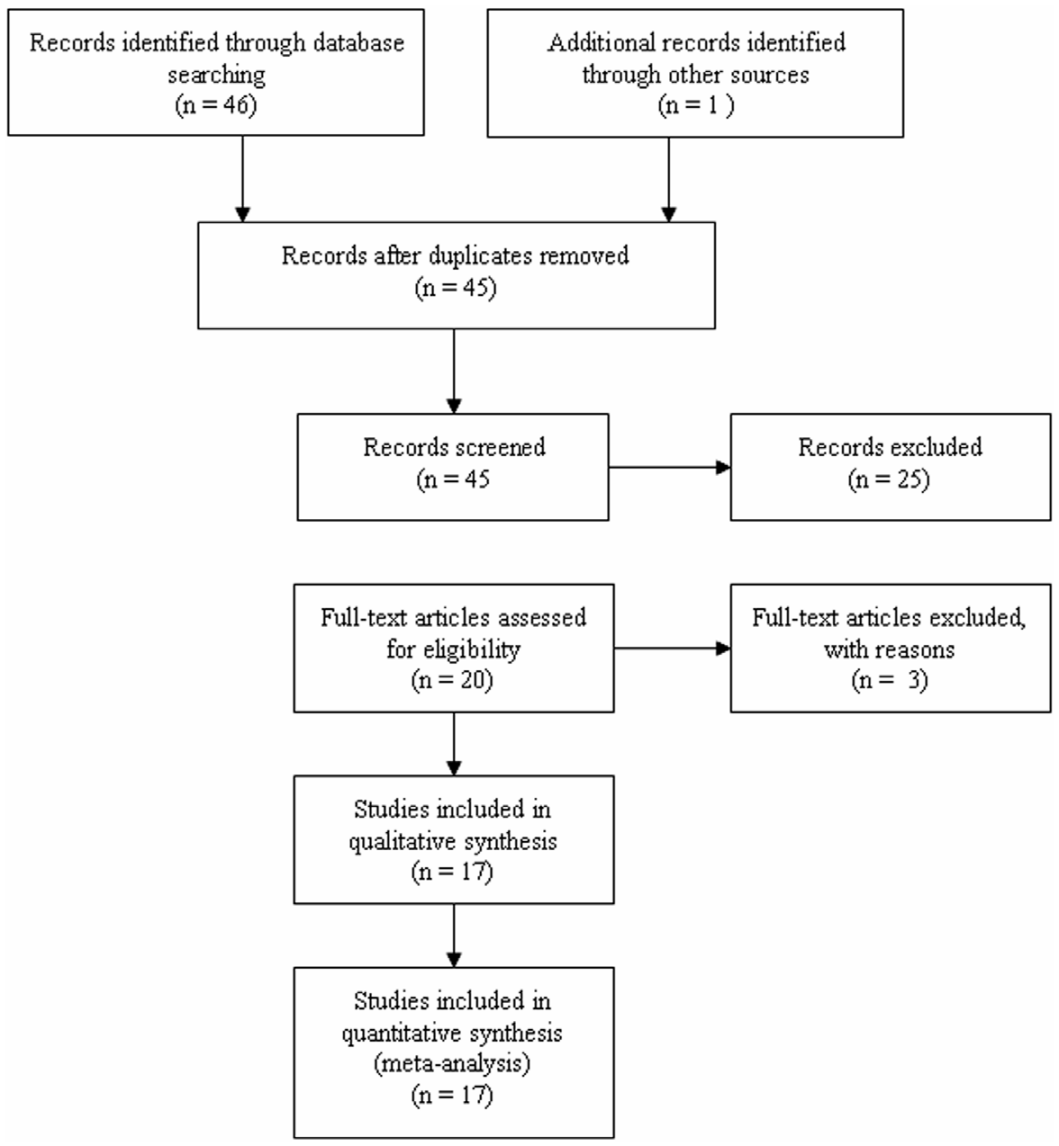
Flow chart of study selection based on the inclusion and exclusion criteria.

47 relevant studies concerning *VDR* or aggrecan *VNTR* polymorphisms and IDD risks were identified. Of these, 25 studies were excluded by reading titles and abstracts. One study investigated other aggrecan polymorphisms rather than *VNTR* [26]; two studies were duplicates [27], [28], and another two studies were excluded for lacking data for pooling [20], [29]. Thus, 17 studies met the inclusion criteria (**Fig. 1**). Among them, a total of 9 studies [10]–[14], [21], [30]–[32], 5 studies [11], [21], [30], [33], [34], 3 studies [13], [14], [31], and 7 studies [11], [16]–[19], [35], [36], were finally included in the meta-analyses for the associations between the *VDR TaqI, FokI, ApaI* or aggrecan *VNTR* polymorphisms and the risk of IDD, respectively. For the *VDR TaqI, FokI, or ApaI* polymorphisms, 5, 1, and 3 studies examined individuals of Asian descent, while the remaining studies recruited Caucasians. For the aggrecan polymorphism, 4 and 3 studies were on Caucasians and Asians, respectively. All the included studies used blood samples for DNA extraction. Magnetic resonance images (MRI) was used for the detection of IDD in almost all the studies, while computed tomography (CT) was used in one study [13]. For the studies about *VDR TaqI, FokI, or ApaI* polymorphisms, genotype distribution in control groups were in HWE except for one study for *TaqI* polymorphism [21], one study for *FokI* polymorphism [33]. For aggrecan polymorphism, no studies included provided the genotypes of each subjects, thus we only compared the difference of allele distribution (≦25 *vs.* >25; ≦23 *vs.* >23). The detailed characteristics of the included studies are shown in the **Table 1** **and** **2**.

**Table 1:** Characteristics of individual studies for associations between VDR polymorphisms and IDD risks.

Authors	Year	Country	Ethnicity	Gender	Age^a^ (Year)	Genotypes distribution						*P*_HWE_ c
						Case^b^			control^b^			
						11	12	22	11	12	22	
***TaqI*** ** (rs731236)**												
Chen	2012	China	Asian	Both	40.3	0	2	79	1	14	86	0.617
Eskola	2010	Danmark	Caucasian	Both	13.1	9	28	29	23	74	57	0.898
Yuan	2010	China	Asian	Both	43.63	0	22	156	0	28	256	0.382
Eser	2010	Turkey	Caucasian	NA	20–30	NA		NA	NA			
Cheung	2006	China	Asian	Both	18–55	1	33	354	0	8	183	0.768
Noponen-Hietala	2003	Filand	Caucasian	Both	48.5	6	11	12	11	19	26	0.044
Oishi	2003	Japan	Asian	Women	73.2	0	8	31	0	5	16	0.536
Kawaguchi	2002	Japan	Asian	Both	22	0	37	79	0	17	72	0.319
Jones	1998	Australia	Caucasian	Both	69.5	OR, 95%CI: 0.47 (0.19–1.16)						NA
***FokI*** ** (rs2228570)**												
Kelempisioti	2011	Filand	Caucasian	Both	40.3	12	51	18	17	48	36	0.032
Eskola	2010	Danmark	Caucasian	Both	13.1	9	28	29	23	74	57	0.898
Eser	2010	Turkey	Caucasian	NA	20–30	NA		NA	NA			
Chen	2007	China	Asian	Both	40.3	12	51	18	17	48	36	0.883
Noponen-Hietala	2003	Filand	Caucasian	Both	48.5	6	12	11	5	26	25	0.630
***ApaI*** ** (rs7975232)**												
Chen	2012	China	Asian	Both	40.3	44	28	9	43	46	12	0.945
Yuan	2010	China	Asian	Both	43.6	58	100	20	128	129	27	0.500
Kawaguchi	2002	Japan	Asian	Both	22	51	48	17	41	39	9	0.951

a the mean age and/or the range of age;

b 11,12,22 represent tt, Tt, TT for *TaqI*(rs731236), ff, Ff, FF for *FokI* (rs2228570), and aa, Aa, AA for *ApaI* (rs7975232), respectively.

c *p* for Hardy–Weinberg equilibrium test in controls;

“NA” means that the data were not available.

**Table 2:** Characteristics of individual studies for association between aggrecan *VNTR* polymorphism and IDD risk.

Authors	Year	Country	Ethnicity	Gender	Age^a^	Alleles (most common one)^b^	Case allels^C^		Control allels^C^	
							≦25	>25	≦25	>25
Kim	2011	Korean	Asian	Both	<40	21;22;23;24;25;26;27;28;33;36 (27)	22	64	3	21
Eser	2011	Turkey	Caucasian	Both	22.3(20–30)	13;21;22;25;26;27;28;29;32 (28)	27	73	19	81
Mashayekhi	2010	Iran	Caucasian	Both	36(28–52)	18;19;20;21;22;23;24;25;26;27;28;29 (27)	33	38	20	88
Eser	2010	Turkey	Caucasian	Men	20–30	13;19;21;22;25;26;27;28;29;32;33 (27)	73	227	55	245
Cong	2010	China	Asian	Men	36.0(14–49)	18;19;20;21;22;23;24;25;26;27;28;29;30;31;32;33 (27)	70	70	92	162
Solovieva	2007	Filand	Caucasian	Men	44(41–46)	21;22;24;25;26;27;28;29;32 (26)	11	19	47	185
Kawaguchi	1999	Japan	Asian	Women	21.3(20–29)	18;21;22;25;26;27;28;29 (27)	11	53	5	59

a the mean age and/or the range of age;

b the variable numbers of tandem repeat(*VNTR*) alleles detected, and the most common form.

c the *VNTR* numbers.

### Quantitative Data Synthesis

**Figure 2:**
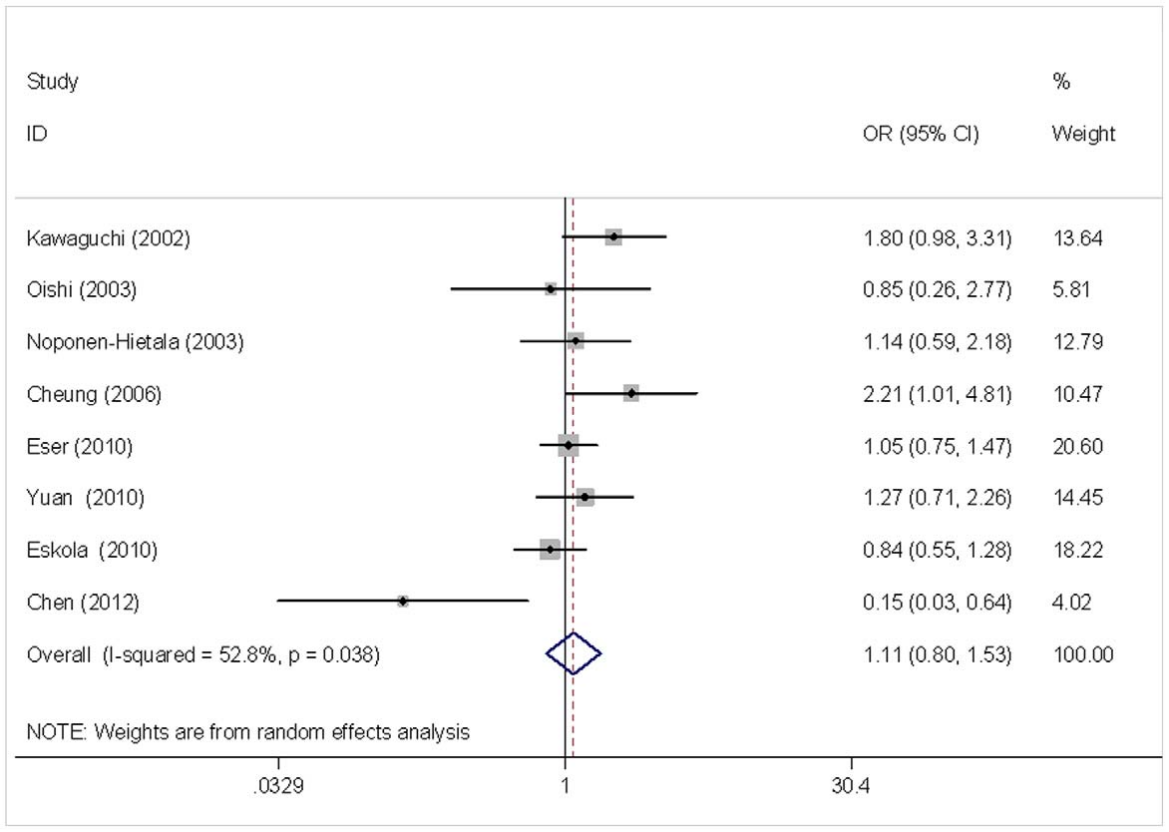
Meta-analysis for *VDR TaqI* polymorphism and the risk of IDD (t *vs.*T). Each study was shown by a point estimate of the effect size (OR) (size inversely proportional to its variance) and its 95% confidence interval (95%CI) (horizontal lines). The white diamond denotes the pooled OR.

Results of pooled analysis on the associations between *VDR* (*TaqI, FokI, ApaI*) polymorphisms and the risk of IDD are shown in **Table 3**. Overall, the combined results showed no significant association between *VDR TaqI* polymorphism and the risk of IDD (t *vs.* T: OR = 1.109, 95%CI 0.803–1.533) (**Fig. 2**). Subgroup analysis stratified by ethnicity, age, and sex, revealed that no associations existed in Caucasians (OR = 0.982, 95%CI 0.769–1.255) or Asians (OR = 1.137, 95%CI 0.599–2.158), in subjects with age >40 (OR = 1.161, 95%CI 0.773–1.742) or ≦40 (OR = 0.928, 95%CI 0.546–1.576), and in women (OR = 0.787, 95%CI 0.505–1.228) or men (OR = 1.172, 95%CI 0.715–1.918). No significant associations were found in genotype contrasts (tt/Tt *vs.* TT: OR = 0.991, 95%CI 0.617–1.591), and the subgroup analysis further confirmed the irrelevance between the genotypes and the risk of IDD. The results were not altered after excluding the study deviated from HWE, further confirming the null association between *VDR TaqI* polymorphism and the risk of IDD. However, when we excluded the studies by Chen *et al.*, in which the 95%CI did not overlap the lines of the pooling results, a significant association was found in Asians (t *vs.* T: OR = 1.568, 95%CI 1.108–2.219).

**Table 3:** Summary of ORs for various genetic contrasts on the associations between *VDR* polymorphisms and the risks of IDD.

	Sub-group	Test of association			Test of heterogenecity	
		OR	95%CI	Statistical model	*I* ^2^ (%)	*p value* a
***TaqI***						
t *vs.* T	Caucasian	0.982	0.769–1.255	FEM	0.0	0.649
	Asian	1.137	0.599–2.158	REM	66.2	0.019
	>40	1.161	0.773–1.742	FEM	0.0	0.830
	≦40	0.928	0.546–1.576	REM	72.3	0.013
	Women	0.787	0.505–1.228	FEM	0.0	0.549
	men	1.172	0.715–1.918	FEM	0.0	0.829
	Both	1.160	0.688–1.954	REM	68.0	0.014
	all	1.109	0.803–1.533	REM	52.8	0.038
Tt/tt *vs.* TT	Caucasian	0.754	0.489–1.162	FEM	7.4	0.340
	Asian	1.158	0.595–2.253	REM	66.0	0.019
	>40	1.003	0.685–1.470	FEM	0.0	0.448
	≦40	1.135	0.375–3.431	REM	83.7	0.000
	Women	0.770	0.453–1.309	FEM	0.0	0.424
	men	1.211	0.658–2.226	FEM	0.0	0.849
	Both	0.949	0.424–2.124	REM	75.3	0.003
	all	0.991	0.617–1.591	REM	62.2	0.010
***FokI***						
f *vs.* F		0.929	0.779–1.109	FEM	25.7	0.250
ff *vs.* FF		1.146	0.719–1.826	FEM	0.0	0.467
Ff/ff *vs.* FF		1.012	0.621–1.649	REM	60.7	0.054
***ApaI***						
a *vs.* A		0.914	0.649–1.288	REM	60.1	0.082
aa *vs.* AA		0.757	0.477–1.202	FEM	0.0	0.379
aa *vs.* AA/Aa		0.924	0.516–1.653	REM	74.7	0.019

a *p* value for heterogeneity based on Q test; FEM, fixed effect model. REM, random effect model.

The pooled results on the associations between *VDR* (*FokI and ApaI*) polymorphisms and the risks of IDD were similar to those of VDR *TaqI* and IDD risk. Overall, no significant association was found between *VDR FokI* polymorphism and IDD risk (f *vs.* F: OR = 0.929, 95%CI 0.779–1.109; ff *vs.* FF: OR = 1.146, 95%CI 0.719–1.826; Ff/ff *vs.* FF: OR = 1.012, 95%CI 0.621–1.649). Similarly, no significant association was found between *VDR ApaI* polymorphism and IDD risk (a *vs.* A: OR = 0.914, 95%CI 0.649–1.288; aa *vs.* AA: OR = 0.757, 95%CI 0.477–1.202; aa *vs.* Aa/AA: OR = 0.924, 95%CI 0.516–1.653). As limited studies were included for the above two association investigation, we did not perform subgroup analysis.

**Figure 3:**
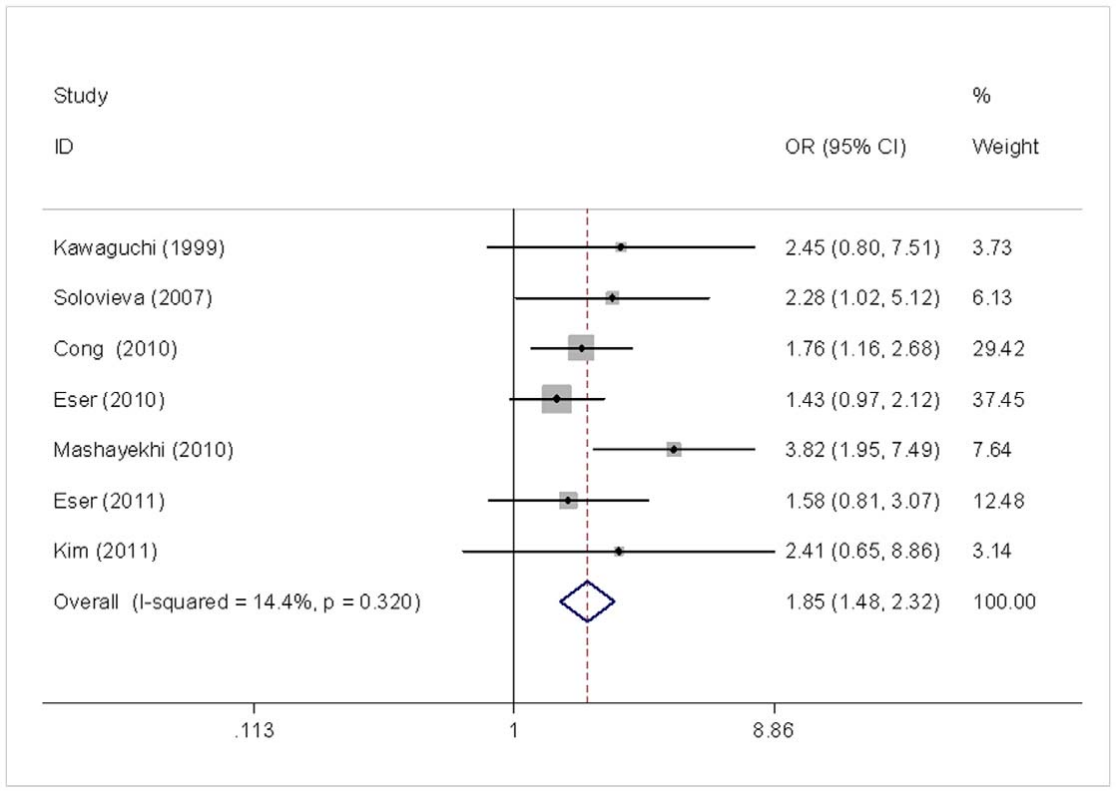
Meta-analysis for aggrecan *VNTR* polymorphism and the risk of IDD (≦25 *vs.* >25). Each study was shown by a point estimate of the effect size (OR) (size inversely proportional to its variance) and its 95% confidence interval (95%CI) (horizontal lines). The white diamond denotes the pooled OR.

Results of pooled analysis on the associations between aggrecan *VNTR* polymorphism and the risk of IDD are shown in **Table 4**. In contrast to the null association between *VDR* polymorphisms and the risk of IDD, a significant association was observed between aggrecan *VNTR* polymorphism and the risk of IDD. The alleles with shorter *VNTR* length was found to significantly increase the risk of IDD (≦25 *vs.* >25: OR = 1.850, 95%CI 1.477–2.318; ≦23 *vs.* >23: OR = 1.955, 95%CI 1.41–2.703) (**Fig. 3**). Significant association was also observed in Caucasians (≦25 *vs.* >25: OR = 2.006, 95%CI 1.468–2.450; ≦23 *vs.* >23: OR = 2.917, 95%CI 1.450–3.329) as well as in Asians (≦25 *vs.* >25: OR = 1.887, 95%CI1.298–2.744; ≦23 *vs.* >23: OR = 1.618, 95%CI 0.960–2.727). Subgroup analysis stratified by gender and age also confirmed the above results.

**Table 4:** Summary of ORs for various genetic contrasts on the association between aggrecan *VNTR* polymorphism and IDD risk.

	Sub-group	No. of studies	Test of association				Test of heterogenecity	
			OR	95%CI	*P* value	Statistical model	*I* ^2^ (%)	*p valu* a
≦25 *vs.* >25	Caucasian	4	2.006	1.468–2.450	0.003	REM	54.2	0.088
	Asian	3	1.887	1.298–2.744	0.001	FEM	0.0	0.800
	>30	3	2.199	1.591–3.040	0.000	FEM	45.6	0.159
	≦30	3	1.537	1.112–2.124	0.009	FEM	0.0	0.673
	Women	1	2.449	0.799–7.508	0.117	FEM	—	—
	men	3	1.636	1.248–2.145	0.000	FEM	0.0	0.549
	Both	3	2.426	1.558–3.778	0.000	FEM	40.4	0.187
	All	7	1.850	1.477–2.318	0.000	FEM	14.4	0.320
≦23 *vs.* >23	Caucasian	3	2.197	1.450–3.329	0.000	FEM	0.0	0.494
	Asian	3	1.618	0.960–2.727	0.071	FEM	0.0	0.747
	>30	3	1.986	1.271–3.103	0.003	FEM	23.3	0.271
	≦30	2	1.845	1.137–2.992	0.013	FEM	0.0	0.924
	Women	1	1.723	0.394–7.535	0.470	FEM	—	—
	men	3	1.691	1.157–2.471	0.007	FEM	0.0	0.966
	Both	2	3.241	1.590–6.606	0.001	FEM	0.0	0.830
	All	6	1.955	1.414–2.703	0.000	FEM	0.0	0.710

a *p* value for heterogeneity based on Q test; FEM, fixed effect model. REM, random effect model.

### Influence Analysis and Cumulative Analysis

After excluding studies that deviated from HWE in controls, and those in which 95%CI did not overlap the lines of the pooling results, no other studies were found to significantly influence the pooled effects in each genetic model. In the cumulative meta-analysis, no particular time trend was found in the summary estimate.

### Publication Bias

**Figure 4:**
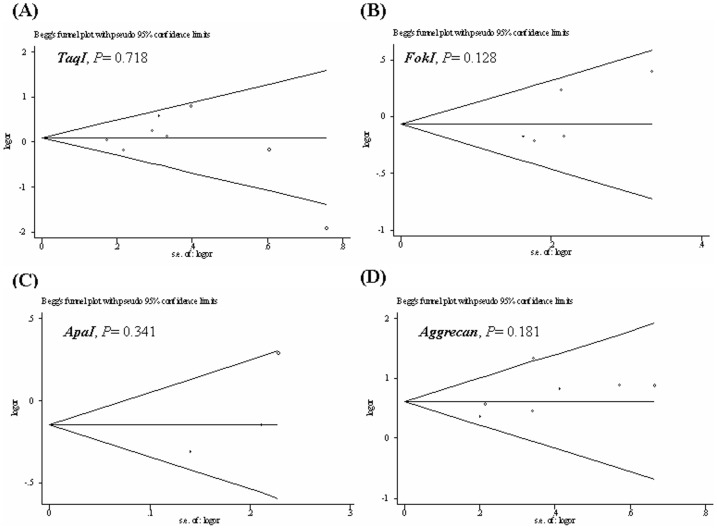
Begg's funnel plot with the Egger's test for publication bias of *VDR* (*TaqI, FokI, ApaI*) and aggrecan *VNTR* polymorphisms and the risk of IDD. The horizontal line in the funnel plot indicates the fixed-effects summary estimate, whereas the diagonal lines pseudo-95% CI limits about the effect estimate. In the absence of publication bias, studies will be distributed symmetrically above and below the horizontal line.

Funnel plots were generated to assess publication bias. The Egger's test was performed to statistically evaluate funnel plot symmetry. The results suggested no publication bias for the association of the *VDR* (*TaqI, FokI, or ApaI*) and aggrecan *VNTR* polymorphisms and the risk of IDD (*P*_Egger test_ = 0.718, 0.128, 0.341, and 0.181, respectively) (**Fig. 4**).

## Discussion

IDD was traditionally regarded as a result of mechanical overloading and senescence; however, recent studies have showed that genetic factors may play a crucial role [35]. In the past few decades, many gene polymorphisms including collagen, interleukins, matrix degrading enzymes, *VDR*, and aggrecan, have been shown to be related with the risks of IDD [6]. *VDR* is the firstly reported gene associated with IDD risk in a study of monozygotic twins in Finns with *FokI* and *TaqI* genotypes [7], while aggrecan *VNTR* polymorphism is a recently widely studied polymorphism for the risk of IDD. Unfortunately, conflicting results are obtained ranging from strong links to no association. The divergent results regarding the effects of these genetic polymorphisms upon IDD risk may be attributed to the differences in racial origin of the population, the age, and the occupation of the subjects. Because of the above-mentioned conflicting results from relatively small studies underpowered to detect the effects, a meta-analysis should be an appropriate approach to obtain a more definitive conclusion.

To the best of our knowledge, this is the first meta-analysis addressing the associations between *VDR* (*TaqI, FokI, ApaI*) and aggrecan *VNTR* polymorphisms and the risks of IDD. In this study, a total of 9, 5, 3, and 7 studies were finally included in the analyses for the association between the *VDR TaqI, FokI, ApaI* or aggrecan *VNTR* polymorphisms and the risks of IDD, respectively. The combined results showed that none of the three *VDR* polymorphisms were significantly associated with the IDD risk. Subgroup analysis stratified by ethnicity, age, and sex, also revealed no association, although a significant association was found in Asians (t *vs.* T: OR = 1.568, 95%CI 1.108–2.219) when excluding one study, in which the 95%CI did not overlap the lines of the pooling results. In contrast, aggrecan *VNTR* polymorphism was found to be significantly associated with the risk of IDD. The alleles of shorter *VNTR* length was found to significantly increase the risk of IDD (≦25 *vs.* >25: OR = 1.850, 95%CI 1.477–2.318; ≦23 *vs.* >23: OR = 1.955, 95%CI 1.41–2.703). Subgroup analysis stratified by ethnicity, gender and age also confirmed the above results. After excluding studies that deviated from HWE in controls, no other studies were found to significantly influence the pooled effects in each genetic model. Cumulative meta-analysis showed that no particular time trend existed in the summary estimate. Furthermore, no potential publication bias was detected by funnel plots and Egger's regression test. These data indicated the robustness of the summary estimate derived from this study.

Aggrecan is the major proteoglycan of the disk, which is responsible for maintaining tissue hydration through the osmotic pressure provided by its constituent chondroitin (CS) and keratin sulfate chasins (KS) [37]. The human aggrecan gene possesses a variable number tandem repeat, *VNTR*, polymorphism in the part of exon 12 encoding the CS1 domain [15]. Alleles have been identified with CS1 repeat numbers ranging from 13 to 33, with the most common alleles containing 26, 27, or 28 repeats [19]. It appears logically that individuals possessing the shortest *VNTR* numbers have the lowest number of CS chains on their aggrecan molecules, and this configuration may result in impaired aggrecan function. Although the association between aggrecan *VNTR* polymorphism and risk of IDD was not found in some studies [20], this meta-analysis provided strong evidence for the above association. The alleles with shorter *VNTR* repeats were overexpressed in IDD patient than control subjects. As no studies provided the genotypes of each participant, thus we did not compare the distribution of genotypes between case and control groups.

*VDR* is a steroid nuclear receptor, better known to have an important role in normal bone mineralization and remodeling. *VDR* expression was reported in chondrocytes and is thought to be involved in differentiation, proliferation, and maturation of cartilage [38]. In addition, vitamin D has been shown to influence proteoglycan synthesis [39]. Polymorphisms in *VDR* gene could influence the stability of the mRNA and vitamin D expression [10]. Although several studies have shown that *VDR* polymorphisms were associated with the risks of IDD, the current meta-analysis did not find any significant association between the three polymorphisms, *FokI* (rs2228570), *TaqI* (rs731236), and *ApaI* (rs7975232), and the IDD risks. However, after scrutiny of the included studies, we could find that most of the studies included for the analysis of aggrecan gene recruited subjects in the absence of other risk factors such as obesity, smoking, heavy physical occupations [11], [16], [18], [35], which were rarely mentioned in the studies for the analysis of *VDR* gene polymorphisms. Thus, it could be speculated that the potential association between *VDR* polymorphisms and IDD may be obscured by some environmental factors. Furthermore, *VDR* polymorphisms have been reported to be significantly associated with the multilevel and severe forms of IDD [11], [31]. Thus, the associations between *VDR* gene polymorphisms and the risks of IDD could not be excluded.

Despite the clear strengths of our study such as the larger sample size comparing with the previous individual ones, it does have some limitations. First, the present meta-analysis was based primarily on unadjusted effect estimates and CIs (since most studies did not provide the adjusted OR and 95%CI controlling for potential confounding factors), thus the effect estimates were relatively imprecise. If individual data were available, adjusted ORs could be obtained to give a more precise analysis. Second, it has been well known that IDD is a multifactor disease, however, the effects of gene-gene and gene-environment interactions were not addressed in this meta-analysis, and thus the potential roles of the above gene polymorphisms may be masked or magnified by other gene-gene/gene-environment interactions. Thirdly, although the funnel plot and Egger's test showed no publication bias, selection bias may also exist because only published studies in English or Chinese were retrieved.

In summary, the current meta-analysis systematically analyzed the associations between *VDR* (*TaqI, FokI, ApaI*) and aggrecan *VNTR* polymorphisms and the risks of IDD. The combined results clearly showed that the alleles with shorter *VNTR* length significantly increased the risk of IDD in Caucasians as well as in Asians. In contrast, none of the *VDR* (*TaqI, FokI, ApaI*) gene polymorphisms were significantly associated with the development of IDD. Since potential confounders could not be ruled out completely, further studies are needed to confirm these results.

## Supporting Information

PRISMA Checklist S1 PRISMA Checklist.
(DOC)
